# Analysing the impact of complex multimorbidity on health-related quality of life

**DOI:** 10.1007/s11136-025-04120-9

**Published:** 2026-01-09

**Authors:** Sharon Walsh, Paddy Gillespie, Anna Hobbins, Ciaran O’Neill, Caroline McCarthy, Frank Moriarty, Barbara Clyne, Fiona Boland, Susan M. Smith

**Affiliations:** 1 Irish Centre for Social Gerontology, J.E. Cairnes School of Business and Economics, University of Galway, University Road, Galway, Ireland; 2 Health Economics and Policy Analysis Centre, J.E. Cairnes School of Business and Economics, University of Galway, University Road, Galway, Ireland; 3 School of Medicine, Dentistry and Biomedical Sciences, Queens University Belfast, University Road, Belfast, Ireland; 4 RCSI University of Medicine and Health Sciences, 123 St Stephen’s Green, Dublin 2, Ireland; 5 Discipline of Public Health and Primary Care, Trinity College Dublin, College Green, Dublin 2, Ireland

**Keywords:** Complex multimorbidity, Health-related quality of life, Multivariate ordered probit model, EQ-5D-5L

## Abstract

**Purpose:**

This paper presents independent associations between complex multimorbidity and health-related quality of life using the EQ-5D-5L instrument**s**. Identifying the decrements in utility associated with complex multimorbidity is of value for economic evaluation and health technology assessment.

**Methods:**

Data from the population normative dataset from the Irish EQ-5D-5L study were combined with baseline data from the SPPiRE (Supporting Prescribing in Older Adults with Multimorbidity in Irish Primary Care) randomised controlled trial. The trial included an Irish cohort aged 65+ with complex multimorbidity. For the analysis, the estimation sample consisted of 364 individuals from the SPPiRE complex multimorbidity sample, along with 116 individuals aged 65+ from the general population who did not report having any serious illness. A multivariate ordered probit regression model was used to estimate the independent associations between complex multimorbidity and the five EQ-5D-5L dimensions.

**Results:**

Complex multimorbidity was independently associated with a lower probability of reporting no problems for all five EQ-5D-5L dimensions, and a higher probability of reporting the most extreme response for all five dimensions. The loss in health utility associated with complex multimorbidity was estimated to be − 0.506 (95% CI − 0.567, − 0.445) relative to those aged 65 years and over with no serious illness. The negative health impact was most pronounced for pain/discomfort and anxiety/depression.

**Conclusion:**

This study reported the associative impacts of complex multimorbidity for all EQ-5D-5L dimensions and for health utility overall. The findings highlight the potential impact on health-related quality of life of interventions that can prevent or delay the onset and progression of multimorbidity.

**Supplementary Information:**

The online version contains supplementary material available at 10.1007/s11136-025-04120-9.

## Plain language summary

Multimorbidity, is the co-existence of two or more chronic conditions in an individual, including physical and mental health conditions, non-communicable diseases, and long-term infectious diseases. It is a growing concern for policy makers for several reasons. Firstly, it can have a significant impact on the individual, in terms of their quality of life, psychological well-being and physical function. In addition, it has an impact on the health system, as individuals with multimorbidity tend to require more complex care, involving multiple medical specialties. In this context, this paper provides important estimates of the impact of multimorbidity on health-related quality of life. We find that having multimorbidity leads to a significant decrease in health-related quality of life across five dimensions - mobility, self-care, usual activities, pain/discomfort and anxiety/depression. This means that if we can develop new models of care that delay or prevent the onset and progression of multimorbidity, it is likely to result in significant health benefits for the individual, and by extension, the health system.

**Supplementary Information:**

The online version contains supplementary material available at 10.1007/s11136-025-04120-9.

## Introduction

Multimorbidity, is generally defined as the co-existence of two or more chronic conditions in an individual, including physical and mental health conditions, non-communicable diseases, and infectious diseases of long duration [1, 2]. The estimated prevalence of multimorbidity in adults is 33%, with prevalence rising to over 50% in those aged 60 and over [3, 4]. Thus, while multimorbidity affects adults of all ages, it is progressively more common with age, which is likely driven by a combination of improvements in survival from acute and chronic conditions, combined with changes in lifestyle risk factors such as physical inactivity and obesity [2, 5–7]. Thus, as countries globally experience population aging, the prevalence of multimorbidity is expected to rise in the coming years [8, 9]. More recently within this context, and of particular relevance to this study, the concept of complex multimorbidity has emerged as a key focus. Complex multimorbidity is based on the principle that some types of multimorbidity may be more difficult to manage than others, both for the healthcare system and the patients themselves [10]. For instance, some patients will have conditions that have little or no impact on their health or quality of life, whereas others will be severely impacted [10]. To identify and prioritise those with higher needs, the concept of ‘complex multimorbidity’ has emerged. While a standardised and universally agreed upon definition of complex multimorbidity does not currently exist, extreme polypharmacy, defined by the number of prescription medications (typically over 10), has emerged as one such indicator of complex multimorbidity status [11, 12].

Thus, with global prevalence rising, multimorbidity has emerged as a significant public health concern. One aspect of this relates to its associated impact on function and health-related quality of life (HRQoL), as well as depression and polypharmacy [13–18]. Indeed, studies employing a range of methods and outcome measures, including the EQ-5D instruments, have consistently found multimorbidity to be associated with poorer HRQoL and psychological well-being [2, 19–22]. EQ-5D is a generic health measure, which is widely used to measure, compare and value health across disease areas [23]. EQ-5D-5L measures health across five dimensions: *mobility; self-care; usual activities; pain/discomfort and anxiety/depression* [24, 25]. The most recent 5L version of the instrument measures each dimension using five severity levels: *no problems; slight problems; moderate problems; severe problems and extreme problems/unable* [26]. Respondents are asked to indicate their current health state by selecting the appropriate level for each dimension [25].The EQ-5D-5L describes 3,125 distinct health states. For each state, the descriptive system can be combined with preference weights for the health states to produce a health utility index reflecting relative preference for health states [24]. This health utility index score is anchored at 1 for full health, 0 for dead, and less than 0 for states valued as worse than dead [27]. In addition to this,. The Visual Analogue Scale (VAS) measures the respondents overall health on a vertical scale, which goes from ‘the best health you can imagine’ to ‘the worst health you can imagine’, providing a quantitative measure of the patient’s perception of their overall health [25].

In the case of multimorbidity, Wong, Xu [28] analysed population-based EQ-5D-5L data in Hong Kong, and found that HRQoL was significantly and negatively affected by the number of chronic conditions, and the type of condition, particularly involving physical disability. Also employing the EQ-5D-5L, Salari, Henrard [29] assessed the HRQoL of multimorbid older adults with polypharmacy in the year following an acute care hospitalisation, and found that being female, housebound and dependent in daily activities were associated with lower HRQoL. These findings are echoed in a number of other studies employing the EQ-5D-3L [19–21, 30–32] and other measures of HRQoL [14, 33, 34]. In addition to the number of chronic conditions, some studies have also explored the types of conditions that contribute most to quality-of-life decrements [20, 31]. However, a recent Nature Reviews Disease Primer showed that while certain disease clusters are associated with poorer quality of life, few replication studies have been carried out, and the observed clusters are not usually replicable [2].

Beyond the individual impact of multimorbidity, concerns have also been raised regarding the healthcare system impact of increasing multimorbidity prevalence. Although individual diseases dominate healthcare delivery, medical research and medical education, people with multimorbidity require a broader approach [6]. Specifically, health systems and policies with a single disease focus lack the integration and coordination required to treat patients with multimorbidity effectively, which can increase treatment burden [2, 35, 36]. In addition, as the number of conditions rise, there is increased burden on healthcare systems related to increased healthcare utilisation and costs [12, 37–39]. For instance, a systematic review by Lehnert, Heider [40] showed a significant increase in utilisation and costs with each additional chronic condition, with some studies identifying a near exponential relationship, in which expenditures almost doubled with each additional chronic condition [40]. In a more recent systematic review, Soley-Bori, Ashworth [41] examined the impact of multimorbidity on healthcare costs and utilisation in the UK. They found that multimorbidity is associated with increased utilisation and costs across a number of resource categories. Interestingly, the largest impact of multimorbidity related to unplanned, potentially preventable hospitalisations, with up to 14.38 times increased odds for those with four or more conditions compared to those with no conditions [41, 42].

Thus, multimorbidity represents an significant health, economic and societal challenge. While multimorbidity research is increasing, there is a dearth of evidence on the clinical and cost effectiveness of interventions targeting people with multimorbidity, with recent systematic reviews documenting sparse and mixed results for health benefits and value for money [43, 44]. In a health economic evaluation and health technology assessment context, EQ-5D instruments have been identified as preferred tools for generating quality-adjusted life years (QALYs) for the purposes of assessing cost effectiveness and informing resource allocation decision-making [45, 46]. Although some concerns have been raised over the use of generic health measures in multimorbid populations [2], a number of studies have shown that the EQ-5D-3L and EQ-5D-5L are valid and responsive instruments in populations with multimorbidity [2, 47–49].

Given this context, this paper builds on the existing evidence base examining the impact of complex multimorbidity on HRQoL, measured and valued using the EQ-5D-5L. Overall, while previous studies consider the relationship between multimorbidity and HRQoL, some have tended to focus specifically on samples of multimorbid patients [E.g. 29, 31, 50], while others have employed general population surveys, using descriptive analysis [E.g. 19, 20, 28]. One study presented a descriptive comparison of EQ-5D outcomes for multimorbid patients relative to the general population [30]. We aim to add to this literature by employing a novel statistical approach, using a multivariate ordered probit model (MVOP) to estimate the impact of complex multimorbidity on HRQoL. We present results for the independent associations between complex multimorbidity (identified from general practice records) and the five EQ-5D-5L dimensions: *mobility, self-care, usual activities, pain/discomfort and anxiety/depression*. In doing so, we shed light on the magnitude of the impacts for each EQ-5D-5L dimension, and the distribution of impacts across the levels of each dimension. Identifying these decrements in utility associated with complex multimorbidity is of value for health economic evaluation and health technology assessments, especially those based on a modelling approach.

## Methods

### Data sources and estimation samples

Two data sources were combined to generate the estimation sample for this analysis. The first source was the SPPiRE (Supporting Prescribing in Older Adults with Multimorbidity in Irish Primary Care) randomised controlled trial (RCT), which examined the clinical and cost effectiveness of a general practitioner-delivered medication review in reducing polypharmacy and potentially inappropriate prescribing in community-dwelling older patients with complex multimorbidity in primary care [12, 51]. Ethical approval was granted by the Irish College of General Practitioners Research Ethics Committee on 25th September 2015. The trial included a representative sample of 403 participants with complex multimorbidity identified from general practice records, aged over 65 years, who were prescribed 15 or more repeat medications but who were well enough to attend the GP for an in-person SPPiRE review. We employ baseline data collected in 2017, which included demographic, socioeconomic, clinical and resource utilisation variables, in addition to the EQ-5D-5L, which we use to capture HRQoL in individuals with complex multimorbidity. In total, 364 trial participants with complete data were included in the estimation sample.

The second source was the population normative dataset from the Irish EQ-5D-5L study [24, 52]. In 2015–2016, the EQ-5D-5L descriptive system was used to measure the self-reported HRQoL of a representative sample of 1,131 adult residents in Ireland, with the aim of generating EQ-5D-5L population normative data and a value set for Ireland. Ethical approval for the study was granted by the University of Galway Ethics Committee on 30th January 2015. Along with EQ-5D-5L data, this study also collected data on demographics, socioeconomics, and variables capturing experience of serious illness. Notably, while participants were asked if they had a serious illness, the nature of the question posed did not allow for a determination of whether the respondent had one or more chronic serious illnesses at the time of study.^1^ In total, 130 individuals aged 65 years and over, reported having experience of a serious illness.

To construct our estimation sample, we first restricted the population normative dataset to those aged 65 and over, to allow for comparison with the SPPiRE complex multimorbidity sample. For the population normative survey, individuals were asked “Have you experienced serious illness”, with a binary yes/no response. For individuals who answered “yes” to this question, we cannot determine whether they have multimorbidity or not. To reduce noise in the estimates, we remove these individuals from the population normative sample. Therefore, the final estimation sample included 364 individuals with complex multimorbidity from the SPPiRE multimorbidity sample (39 respondents were dropped due to non-response on key variables of interest), along with 116 individuals aged 65+ from the population normative dataset who did not report having a serious illness.^2^

### Statistical analysis

To analyse the association between complex multimorbidity status and HRQoL, we estimate a MVOP regression model of all five EQ-5D-5L health dimensions simultaneously, following the approach adopted by Henry and Cullinan [53]. The multivariate ordered probit regression approach may be viewed as a natural extension of the univariate ordered probit model in a seemingly unrelated regression framework [54]. The correlation matrix presented in Table 3, shows a positive correlation in errors. This supports the use of an MVOP model and implies that respondents with a higher propensity to report a problem in one health dimension are also more likely to report a problem in another dimension. Thus, the model accommodates both observable and unobservable common factors, through the independent variables and the error terms included in the equations [55].

The key independent variable in this analysis is complex multimorbidity status. Within the SPPiRE trial, significant polypharmacy was used as a proxy marker for complex multimorbidity. Specifically, complex multimorbidity was defined as being prescribed 15 or more regular medicines. The use of significant polypharmacy as a proxy for complex multimorbidity is considered a valid approach in the literature [11, 12]. Therefore, we do not observe which chronic conditions people are experiencing. Rather, individuals who meet this threshold (15 or more regular medications) were identified by general practitioners as having complex multimorbidity. In our analysis, we generate a complex multimorbidity dummy variable, equal to one for those with complex multimorbidity (SPPiRE sample) and equal to zero for those without complex multimorbidity (normative sample). We also included available observable personal and socioeconomic characteristics as controls. These included age, gender, employment status, education, location (urban/rural), medical card status (means-tested free access to general practitioner services and prescription medicines), and private health insurance status. We present findings in two ways. First, we present average partial effect estimates in the form of the impact of complex multimorbidity status on the probability of reporting each level for the EQ-5D-5L dimensions. Second, we combine the estimated partial effects with the published utility decrements from the Irish EQ-5D-5L value set to estimate the health utility losses associated with complex multimorbidity for each dimension individually and for health overall [52]. Details of the Irish value set are provided in the appendix.

In sensitivity analysis, we present results for alternative estimation samples including the full EQ-5D-5L population norm sample, and the full EQ-5D-5L population norm sample aged 65 and over. We also present results for univariate models controlling for complex multimorbidity status only, and for a series of ordered probit models estimated independently for each of dimension. Unlike the MVOP, these models do not control for potential correlations in the errors. All analyses were performed using Stata 18.0 [56] and Microsoft Excel, and statistical significance was considered at the 0.05 level using 95% Confidence Intervals (CI).

## Results

Definitions and sample descriptive statistics for the variables used in the analysis are presented in Table 1. Regarding the EQ-5D-5L data, there are notable differences in HRQoL between the complex multimorbidity sample and the population normative sample. For instance, within the normative general population sample, 78.34% of the general population reported having no problems with mobility, while 64.66% of those aged 65 years and over, with no serious illness, reported having no problems with mobility. This compares to 15.93% of those in the complex multimorbidity sample. The equivalent statistics were 93.72% and 93.97% versus 55.22% for the self-care dimension, 80.81% and 84.48% versus 24.18% for usual activities, 59.50% and 50.86% versus 9.07% for pain/discomfort, and 80.81% and 88.79% versus 41.76% for anxiety/depression. The difference in HRQoL between the groups is also highlighted when we compare the health utility index scores and Visual Analogue Scale (VAS) scores. Specifically, when we apply the Irish value set to the normative general population sample, we find that the general population had an average index score of 0.889, while the general population aged 65 and over with no serious illness, had an average index score of 0.890. This compares to the complex multimorbidity sample, with an average index score of 0.482. A similar pattern is found regarding the VAS scores. The general population had an average VAS score of 79.97, while those aged 65 and over, with no serious illness, had an average VAS score of 83.66. This compares to an average VAS score of 59.44 within the SPPiRE multimorbidity sample.

**Table 1:** Variable definitions and sample descriptive statistics. *Source*: Analysis of data from the Irish EQ-5D-5L Survey, 2015/16 [65], and data from the SPPiRE randomised controlled trial [12]

Variable name	Variable description	Irish population normative sample		SPPiRE multimorbidity sample
		Full Sample	Aged 65 + Sample, no serious illness	
		N (%) / Mean (SD)	N (%) / Mean (SD)	N (%) /Mean (SD)
*Dependent variables: EQ-5D health dimensions*				
Mobility	None	886 (78.34)	75 (64.66)	58 (15.93)
	Slight	143 (12.64)	25 (21.55)	76 (20.88)
	Moderate	77 (6.81)	12 (10.34)	123 (33.79)
	Severe	20 (1.77)	4 (3.45)	93 (25.55)
	Unable	5 (0.44)	0 (0)	14 (3.85)
Self-care	None	1,060 (93.72)	109 (93.97)	201 (55.22)
	Slight	54 (4.77)	7 (6.03)	66 (18.13)
	Moderate	12 (1.06)	0 (0)	62 (17.03)
	Severe	4 (0.35)	0 (0)	22 (6.04)
	Unable	1 (0.09)	0 (0)	13 (3.57)
Usual activities	None	914 (80.81)	98 (84.48)	88 (24.18)
	Slight	127 (11.23)	8 (6.90)	87 (23.90)
	Moderate	62 (5.48)	5 (4.31)	91 (25.00)
	Severe	21 (1.86)	2 (1.72)	59 (16.71)
	Unable	7 (0.62)	3 (2.59)	39 (10.71)
Pain/discomfort	None	673 (59.50)	59 (50.86)	33 (9.07)
	Slight	270 (23.87)	31 (26.72)	78 (21.43)
	Moderate	152 (13.44)	22 (18.97)	139 (38.19)
	Severe	31 (2.74)	3 (2.59)	94 (25.82)
	Extreme	5 (0.44)	1 (0.86)	20 (5.49)
Anxiety/depression	None	914 (80.81)	103 (88.79)	152 (41.76)
	Slight	127 (11.23)	10 (8.62)	99 (27.20)
	Moderate	62 (5.48)	3 (2.59)	92 (25.27)
	Severe	21 (1.86)	0 (0)	14 (3.85)
	Extreme	7 (0.62)	0 (0)	7 (1.92)
EQ-VAS score		79.97 (14.91)	83.66 (11.73)	59.44 (20.91)
EQ-5D-5L index score		0.889 (0.174)	0.890 (0.157)	0.482 (0.373)
*Independent variables*				
Age category	18–24 years	88 (7.78)	0 (0)	0 (0)
	25–34 years	165 (14.59)	0 (0)	0 (0)
	35–44 years	221 (19.54)	0 (0)	0 (0)
	45–54 years	229 (20.25)	0 (0)	0 (0)
	55–64 years	182 (16.09)	0 (0)	0 (0)
	65–74 years	156 (13.79)	82 (70.69)	148 (40.66)
	75–84 years	78 (6.90)	30 (25.86)	170 (46.70)
	85 + years	12 (1.06)	4 (3.45)	46 (12.64)
Gender	Male	426 (37.67)	30 (25.86)	162 (44.51)
Employment status	Full Time Employed	384 (33.95)	4 (3.45)	11 (3.02)
	Home duties	124 (10.96)	8 (6.90)	58 (15.93)
	Unemployed	71 (6.28)	0 (0)	0 (0)
	Student	70 (6.19)	0 (0)	0 (0)
	Retired	255 (22.55)	96 (82.76)	289 (79.40)
	Long-term sick/disability	42 (3.71)	0 (0)	0 (0)
	Part time employed	168 (14.85)	7 (6.03)	0 (0)
	Other	17 (1.50)	1 (0.86)	6 (1.65)
Education	Primary education	86 (7.60)	27 (23.28)	149 (40.93)
	Secondary education	414 (36.60)	52 (44.83)	148 (40.66)
	Higher education	631 (55.79)	37 (31.90)	67 (18.41)
Location	Urban location	656 (58.00)	59 (50.86)	319 (87.64)
Medical card status	Medical card	455 (40.23)	81 (69.83)	357 (98.08)
Private health insurance status	Private health insurance	637 (56.32)	79 (68.10)	127 (34.89)
Serious illness status	Serious illness	375 (33.16)	0 (0)	n/a
No. of observations		1131	116	364

Using the estimation sample, we then estimate the average partial effects and the predicted losses in health utility associated with complex multimorbidity status, and the results are presented in Table 2. For brevity, we only present the partial effects for complex multimorbidity. However, full results are available on request. We find that complex multimorbidity was independently associated with a lower probability of reporting no problems across all five EQ-5D-5L dimensions: *Mobility*: − 0.335 (95% CI − 0.405, − 0.264); *Self Care*: − 0.547 (95% CI − 0.676, − 0.419); *Usual Activities*: − 0.436 (95% CI − 0.515, − 0.356); *Pain/Discomfort*: − 0.263 (95% CI − 0.324, − 0.201); *Anxiety/Depression*: − 0.466 (95% CI − 0.573, − 0.360). Further, complex multimorbidity was independently associated with a higher probability of reporting the most extreme outcome across all five EQ-5D-5L dimensions: *Mobility*: 0.083 (95% CI 0.044, 0.122); *Self Care*: 0.101 (95% CI 0.050, 0.152); *Usual Activities*: 0.217 (95% CI 0.151, 0.283); *Pain/Discomfort*: 0.104 (95% CI 0.061, 0.147); *Anxiety/Depression*: 0.049 (95% CI 0.016, 0.083). The overall loss in health utility associated with complex multimorbidity was estimated at − 0.506 (95% CI − 0.567, − 0.445), of which -0.086 (95% CI − 0.109, − 0.065) was related to *Mobility*, − 0.082 (95% CI − 0.104, − 0.061) was related to *Self Care*, − 0.070 (95% CI − 0.086, − 0.055) was related to *Usual Activities*, − 0.137 (95% CI − 0.171, − 0.106) was related to *Pain/Discomfort*, and − 0.131 (95% CI − 0.163, − 0.095) was related to *Anxiety/Depression.*

**Table 2:** Estimated partial effects for multimorbidity status on EQ-5D-5L dimension level probabilities and health utilities. *Source*: Analysis of data from the Irish EQ-5D-5L Survey, 2015/16 [65], and data from the SPPiRE randomised controlled trial [12]

EQ-5D-5L dimension/ level	Estimates (95% CI) estimation sample
*Mobility*	
None	− 0.335 (− 0.405, − 0.264)
Slight	− 0.088 (− 0.119, − 0.056)
Moderate	0.080 (0.047, 0.112)
Severe	0.259 (0.190, 0.328)
Unable	0.083 (0.044, 0.122)
Estimated health utility loss	− 0.086 (− 0.109, − 0.065)
*Self-care*	
None	− 0.547 (− 0.676, − 0.419)
Slight	0.108 (0.073, 0.143)
Moderate	0.216 (0.147, 0.285)
Severe	0.122 (0.068, 0.176)
Unable	0.101 (0.050, 0.152)
Estimated health utility loss	− 0.082 (− 0.104, − 0.061)
*Usual activities*	
None	− 0.436 (− 0.515, − 0.356)
Slight	− 0.035 (− 0.065, − 0.005)
Moderate	0.102 (0.071, 0.134)
Severe	0.152 (0.106, 0.197)
Unable	0.217 (0.151, 0.283)
Estimated health utility loss	− 0.070 (− 0.086, − 0.055)
*Pain/discomfort*	
None	− 0.263 (− 0.324, − 0.201)
Slight	− 0.130 (− 0.165, − 0.094)
Moderate	0.054 (0.021, 0.087)
Severe	0.235 (0.172, 0.297)
Extreme	0.104 (0.061, 0.147)
Estimated health utility loss	− 0.137 (− 0.171, − 0.106)
*Anxiety/depression*	
None	− 0.466 (− 0.573, − 0.360)
Slight	0.081 (0.050, 0.112)
Moderate	0.263 (0.187, 0.339)
Severe	0.074 (0.034, 0.114)
Extreme	0.049 (0.016, 0.083)
Estimated health utility loss	− 0.131 (− 0.163, − 0.095)
Estimated total health utility loss	− 0.506 (− 0.567, − 0.445)
N	480

This table presents partial effect estimates for *Multimorbidity* from a multivariate ordered probit model (MVOP) of the EQ-5D health dimensions. 95% Confidence Intervals in parenthesis. For the health utility estimates, the 95% CI estimated on the basis of multiplicands of 1000 random draws from the probability distributions for the EQ-5D-5L utility values and the MVOP 5L partial effect estimates

The results from a series of sensitivity analyses are presented in the supplementary materials and generally confirm the base-case analyses. Notably, the overall loss in health utility was estimated at − 0.193 (95% CI − 0.220, − 0.164, n = 1495) for the estimation sample including the full EQ-5D-5L population norm sample, and − 0.339 (95% CI − 0.373, − 0.303, n = 610) for the estimation sample including the full population norm sample aged 65 and over.

## Discussion

Complex multimorbidity represents a health, economic, and societal challenge for policy makers and health and social care systems globally [12–16]. To address the societal and health system challenges associated with complex multimorbidity, evidence on the impact of complex multimorbidity on HRQoL will form an essential input to evidence-based decision making. This paper showed that complex multimorbidity is independently associated with a lower probability of reporting no problems for all five EQ-5D-5L dimensions, and a higher probability of reporting the most extreme response for all five dimensions. The estimated magnitude of health disutility related to complex multimorbidity was − 0.506 (95% CI − 0.567, − 0.445), which is both clinically and statistically significant. This observed health decrement may be driven in part by the fact that the focus of the SPPiRE trial was individuals over the age of 65 years, who were being prescribed 15 or more repeat medicines, which is a measure of both significant polypharmacy and complex multimorbidity [57]. Therefore, our complex multimorbidity sample had a high treatment and disease burden, when compared to other cohorts with multimorbidity [43]. Nevertheless, the disutility that we identified goes to highlight the potential health benefits from interventions that delay or prevent the onset and progression of multimorbidity.

After applying the utility weights elicited from the Irish population and calculating the total utility loss associated with complex multimorbidity for each dimension, the pain/discomfort and anxiety/depression dimensions exhibited the greatest decrements. Notably, these are influenced by both the magnitude of the MVOP coefficients and the magnitudes of the preference weights in the Irish value set. As outlined in Appendix, Table 4, while the MVOP coefficient was greater for pain/discomfort than for anxiety/depression, it was the latter dimension that was valued more highly in the Irish value set, which led to the similar utility loss estimates for each dimension that we observe in our analysis [52]. Nevertheless, this is consistent with the international literature and pose interesting challenges for policy makers. For instance, Mujica-Mota, Roberts [58] and Brettschneider, Leicht [59] showed that mental health problems had a significant adverse effect on HRQoL, while Mujica-Mota, Roberts [58] found that the associations of physical health with HRQoL were stronger when combined with long-term mental health problems. Thus, an integrated treatment approach, which addresses both physical and mental health issues in complex multimorbid patients is likely to be highly beneficial. Interestingly, a recent Cochrane review showed that interventions that addressed depression in multimorbid populations, with depression as a comorbidity, were effective at improving mental health outcomes [60]. However, mental health improvements were much lower for generic interventions that target multimorbidity. This presents a challenge when designing generic interventions that address both physical and mental health. Nevertheless, a recent systematic review showed that exercise therapy improved HRQoL, as well as physical function and depression/anxiety symptoms in people with multimorbidity [17].

Regarding pain/discomfort, the high disutility that we find is echoed in several previous studies, which consider the types of chronic conditions that impact most on HRQoL. For instance, Bao, Xie [20] found that the combination of chronic pain and bone disease was associated with the greatest loss in HRQoL. Similarly, Mujica-Mota, Roberts [58] showed that arthritis and long-term back problems were the physical conditions most strongly associated with reduced HRQoL. At the aggregate level, a recent systematic review showed that while there is a lack of evidence for interventions targeting pain management in people with multimorbidity, there is some evidence in support of interventions targeting osteoarthritis in these individuals [17]. While the relationship between complex multimorbidity and HRQoL was the focus of this study, several other variables were included as controls in the statistical analysis. Of particular note, we found that those with private health insurance had a higher probability of reporting no problems, and a lower probability of reporting problems with mobility, self-care, usual activities and pain/discomfort (See Appendix Table 5). Voluntary private health insurance status is an established indicator of socioeconomic status in Ireland, with those in higher social classes significantly more likely to have private health insurance than those in lower social classes [61, 62]. Thus, this finding reflects the association between socioeconomic status and HRQoL, which has been well established in the literature [e.g. 63, 64].

When considering the results of our analysis, a number of caveats should be borne in mind. First, our data are cross-sectional, and thus our estimates should be considered as independent associations rather than causal effects. Future research should look, where possible, to employ alternative study designs to aid casual inference. The combination of data from two sources to generate the estimation sample, collected at different times and via different study designs and methods, is an important consideration. However, we sought to limit bias, and reduce noise in the estimated relationships, by combining samples that were broadly comparable. Specifically, we excluded those in the normative sample who self-reported as having a serious illness, as it was not possible to determine the nature or extent of these illnesses. Given that these are self-reported data, there is a small possibility that someone with one or more chronic conditions, may not self-identify as having a serious illness. If this were the case, then it may bias our estimates, however, we expect this to be relatively low risk. Nevertheless, some demographic differences are still evident. Specifically, the SPPiRE sample is older than the population normative sample, with 12.64% aged 85 + in the SPPiRE sample, compared to 3.45% in the population normative sample. There are also some socio-economic differences between the two samples. A smaller portion of those in the SPPiRE sample have private health insurance (34.89% versus 68.10% in the population normative sample). They are also less likely to hold a third level education (18.41% versus 31.9% in the population normative sample). These differences are not surprising given that socioeconomic deprivation and lower education levels are associated with a higher prevalence of multimorbidity. Nevertheless, it may limit the generalisability in our findings. In addition, it should be noted that, to be included in the SPPiRE sample, respondents had to be well enough to attend the GP practice in person, which may introduce some bias into our estimate, as those who are very severely unwell are likely to be excluded.

The statistical analysis, while a strength of the study, was constrained by the number of independent variables available for inclusion in our control set. While we control for unobservable factors with the MVOP model, there still exists the potential for bias in our results. That said, the correlation matrix that estimates the relationship between the error terms of the different outcomes were positive and statistically significant. This supports the use of the seemingly unrelated regression approach adopted and implies that respondents with a higher propensity to report a problem in one health dimension also have a higher propensity to report a problem in another dimension. While our key complex multimorbidity status independent variable was identified from general practice records and therefore objective, the outcome measures and other independent variables were self-reported and subjective. Further, the complex multimorbidity status variable was a proxy measure based on the number of regular prescribed medications, and therefore did not provide clarity on the timing, nature or number of chronic conditions. This is a limitation of the study as we were not able to explore the implications of these issues. Further, the variable on serious illness experience in the EQ-5D-5L survey did not provide for clarity on the nature or timing of the serious illness, or if the individual had multiple illnesses: that is, if they were multimorbid.

Despite these caveats, we believe that the analysis and general results and conclusions presented should be of interest to policy makers and health and social care system managers, as it presents further estimates of the impact of complex multimorbidity on HRQoL, as well as the decrements in utility associated with complex multimorbidity. Additionally, the findings will be of use for health economic evaluation modelling for health technology assessment in the context of complex multimorbidity.

## Conclusions

The findings of the present study indicate that the burden experienced by individuals living with complex multimorbidity extend across all five health dimensions captured by the EQ-5D-5L instrument and to health utility overall. The findings add to the evidence base on the application of EQ-5D instruments for multimorbid populations and further highlight the potential health benefits from interventions that prevent the onset and progression of multimorbidity.

## Supplementary Information

Below is the link to the electronic supplementary material.

Supplementary Material 1

## Appendix

See Tables 3, 4 and 5.

**Table 3:** Estimated correlation matrix. *Source*: Analysis of data from the Irish EQ-5D-5L Survey, 2015/16 [65], and data from the SPPiRE randomised controlled trial [12]

	*ε*_1_	*ε*_2_	*ε*3	*ε*_4_	*ε*_5_
*ε*_1_	1				
*ε*_2_	0.642 (0.038)	1			
	(0.560, 0.711)				
*ε*_3_	0.710 (0.029)	0.759 (0.029)	1		
	(0.648, 0.763)	(0.696, 0.811)			
*ε*_4_	0.569 (0.037)	0.479 (0.047)	0.550 (0.039)	1	
	(0.491, 0.637)	(0.383, 0.565)	(0.470, 0.622)		
*ε*_5_	0.285 (0.054)	0.399 (0.054)	0.426 (0.050)	0.304 (0.053)	1
	(0.176, 0.387)	(0.287, 0.499)	(0.324, 0.518)	(0.197, 0.403)	

The table presents the estimated correlation matrix Ω, where ρ ^_jk represents the estimated correlation between error term ε_j and ε_k. Robust standard errors in parentheses and 95% confidence internals

**Table 4:** EQ-5D-5L Irish value set. *Source*: Taken from Hobbins, Barry [52]

Mobility		Self-care		Usual activity		Pain/Discomfort		Anxiety/depression	
None	0	None	0	None	0	None	0	None	0
Slight	0.063	Slight	0.055	Slight	0.049	Slight	0.068	Slight	0.08
Moderate	0.097	Moderate	0.088	Moderate	0.072	Moderate	0.093	Moderate	0.202
Severe	0.215	Severe	0.229	Severe	0.154	Severe	0.373	Severe	0.535
Unable	0.344	Unable	0.287	Unable	0.187	Extreme	0.51	Extreme	0.646

**Table 5:** Estimated partial effects for private health insurance on EQ-5D-5L dimension level probabilities

EQ-5D-5L Dimension/Level	Private Health Insurance Estimates (95% CI)
*Mobility*	
None	0.079 (0.020, 0.138)
Slight	0.021 (0.005, 0.037)
Moderate	− 0.019 (− 0.035, − 0.003)
Severe	− 0.061 (− 0.107, − 0.016)
Unable	− 0.020 (− 0.036, − 0.003)
*Self-care*	
None	0.106 (0.029, 0.184)
Slight	− 0.021 (− 0.037, − 0.005)
Moderate	− 0.042 (− 0.073, − 0.011)
Severe	− 0.024 (− 0.043, − 0.004)
Unable	− 0.020 (− 0.037, − 0.003)
*Usual activities*	
None	0.077 (0.010, 0.145)
Slight	0.006 (− 0.001, 0.013)
Moderate	− 0.018 (− 0.035, − 0.002)
Severe	− 0.027 (− 0.051, − 0.003)
Unable	− 0.038 (− 0.073, − 0.004)
*Pain/discomfort*	
None	0.049 (0.001, 0.097)
Slight	0.024 (0.000, 0.048)
Moderate	− 0.010 (− 0.022, 0.002)
Severe	− 0.043 (− 0.086, − 0.001)
Extreme	− 0.019 (− 0.039, 0.001)
*Anxiety/depression*	
None	− 0.012 (− 0.094, 0.069)
Slight	0.002 (− 0.012, 0.016)
Moderate	0.007 (− 0.039, 0.053)
Severe	0.002 (− 0.011, 0.015)
Extreme	0.001 (− 0.007, 0.010)
N	480

This table presents partial effect estimates from a multivariate ordered probit model (MVOP) of the EQ-5D health dimensions. 95% Confidence Intervals in parenthesis

## Footnotes

1 The wording of the question is included in the supplementary materials.

2 For robustness, we also estimated the models using different estimation samples. Results available in the supplementary materials.
