# Supplementary material for: Analysing the impact of complex multimorbidity on health-related quality of life
Source: Qual Life Res. 2026 Jan 9;35(2):40. doi: 10.1007/s11136-025-04120-9 (PMC12789114; doi:10.1007/s11136-025-04120-9)
Supplement: Supplementary file 1 — Supplementary Material 1 [file 11136_2025_4120_MOESM1_ESM.docx]

**Supplementary Materials**

Serious illness question from the Irish EQ-5D-5L Irish questionnaire


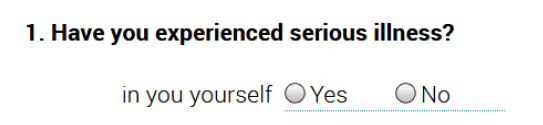


**Table S1:** Estimated partial effects for multimorbidity status on EQ-5D-5L dimension level probabilities and health utilities – MVOP with different estimation samples

| **EQ-5D-5L Domain/ Level** | **Estimates**  **(95% CI)**  **Full**  **Combined Sample** | **Estimates**  **(95% CI)**  **Full Over 65s**  **Estimation Sample** |
| --- | --- | --- |
| **Mobility** |  |  |
| None | -0.217 (-0.263, -0.172) | -0.273 (-0.329, -0.217) |
| Slight | 0.049 (0.035, 0.063) | -0.042 (-0.058, -0.026) |
| Moderate | 0.070 (0.055, 0.086) | 0.086 (0.063, 0.110) |
| Severe | 0.069 (0.053, 0.085) | 0.176 (0.133, 0.219) |
| Unable | 0.029 (0.018, 0.040) | 0.053 (0.029, 0.077) |
| *Estimated health utility loss* | -0.035 (-0.041, -0.028) | -0.062 (-0.075, -0.048) |
| **Self-Care** |  |  |
| None | -0.134 (-0.169, -0.099) | -0.281 (-0.351, -0.211) |
| Slight | 0.048 (0.033, 0.062) | 0.076 (0.054, 0.098) |
| Moderate | 0.045 (0.031, 0.060) | 0.104 (0.071, 0.137) |
| Severe | 0.023 (0.013, 0.032) | 0.056 (0.032, 0.080) |
| Unable | 0.018 (0.009, 0.027) | 0.045 (0.023, 0.068) |
| *Estimated health utility loss* | -0.017 (-0.021, -0.013) | -0.039 (-0.049, -0.030) |
| **Usual Activities** |  |  |
| None | -0.221 (-0.268, -0.174) | -0.306 (-0.368, -0.243) |
| Slight | 0.060 (0.044, 0.076) | 0.003 (-0.009, 0.016) |
| Moderate | 0.065 (0.050, 0.080) | 0.084 (0.062, 0.105) |
| Severe | 0.046 (0.034,0.058) | 0.104 (0.075, 0.132) |
| Unable | 0.050 (0.035, 0.064) | 0.115 (0.079, 0.151) |
| *Estimated health utility loss* | -0.024 (-0.029, -0.020) | -0.044 (-0.053, -0.035) |
| **Pain / Discomfort** |  |  |
| None | -0.290 (-0.347, -0.234) | -0.271 (-0.322, -0.220) |
| Slight | 0.052 (0.036, 0.068) | -0.083 (-0.104, -0.062) |
| Moderate | 0.118 (0.094, 0.142) | 0.087 (0.062, 0.113) |
| Severe | 0.087 (0.068, 0.105) | 0.186 (0.146, 0.227) |
| Extreme | 0.033 (0.021, 0.045) | 0.080 (0.050, 0.111) |
| *Estimated health utility loss* | -0.064 (-0.087, -0.039) | -0.113 (-0.135, -0.089) |
| **Anxiety / Depression** |  |  |
| None | -0.248 (-0.307, -0.188) | -0.320 (-0.390, -0.251) |
| Slight | 0.094 (0.070, 0.119) | 0.075 (0.054, 0.097) |
| Moderate | 0.114 (0.084, 0.143) | 0.174 (0.129, 0.219) |
| Severe | 0.027 (0.016, 0.039) | 0.044 (0.021, 0.067) |
| Extreme | 0.013 (0.005, 0.020) | 0.027 (0.009, 0.045) |
| *Estimated health utility loss* | -0.053 (-0.063, -0.043) | -0.082 (-0.102, -0.062) |
| ***Estimated total health utility loss*** | -0.193 (-0.220, -0.164) | -0.339 (-0.373, -0.303) |
| **N** | 1495 | 610 |

**Notes:** This table presents partial effect estimates for *Multimorbidity* from a multivariate ordered probit model (MVOP) of the EQ-5D health dimensions. 95% Confidence Intervals in parenthesis. For the health utility estimates, the 95% CI estimated on the basis of multiplicands of 1000 random draws from the probability distributions for the EQ-5D-5L utility values and the MVOP 5L partial effect estimates.

**Source:** Analysis of data from the Irish EQ-5D-5L Survey, 2015/16 (59), and data from the SPPiRE randomised controlled trial (60).

**Table S2:** Estimated Partial Effects (Standard Error, P-Value, 95% Confidence Interval) for Multimorbidity Status on EQ-5D-5L Dimension Level Probabilities – Ordered Probit Models

| **EQ5D-5L**  **Dimension** | **EQ-5D-5L**  **Level** | **OPROB**  **without**  **controls** | **OPROB**  **with controls** | **OPROB**  **without**  **controls** | **OPROB**  **With**  **controls** | **OPROB**  **without**  **controls** | **OPROB**  **With**  **controls** |
| --- | --- | --- | --- | --- | --- | --- | --- |
|  |  | *Full Sample* | *Full Sample* | *Over 65s Sample* | *Over 65s Sample* | *Over 65s Sample w/o Serious Illness* | *Over 65s Sample w/o Serious Illness* |
| **Mobility** | **None** | -0.480  (0.012) (0.000)  (-0.504, -0.456) | -0.226  (0.024) (0.000)  (-0.272,-0.180) | -0.329  (0.023) (0.000)  (-0.373, -0.284) | -0.268  (0.029) (0.000)  (-0.324, -0.212) | -0.377  (0.026) (0.000)  (-0.427, -0.327) | -0.330  (0.036) (0.000)  (-0.401, -0.259) |
|  | **Slight** | 0.124  (0.009) (0.000)  (0.107, 0.141) | 0.074  (0.008) (0.000)  (0.058, 0.090) | -0.045 (0.009) (0.000)  (-0.063, -0.027 | -0.042  (0.008) (0.000)  (-0.058, -0.026) | -0.095  (0.016) (0.000)  (-0.127, -0.063) | -0.087  (0.016) (0.000)  (-0.118, -0.055) |
|  | **Moderate** | 0.162  (0.010) (0.000)  (0.143, 0.181) | 0.051  (0.008) (0.000)  (0.036, 0.065) | 0.105  (0.012) (0.000)  (0.082, 0.128 | 0.085  (0.012) (0.000) (0.061, 0.108) | 0.089  (0.017) (0.000) (0.056, 0.123) | 0.078  (0.016) (0.000) (0.046, 0.110) |
|  | **Severe** | 0.143  (0.011) (0.000) (0.122, 0.164) | 0.075  (0.009) (0.000)  (0.058, 0.092) | 0.211  (0.021) (0.000)  (0.169, 0.252) | 0.176  (0.022) (0.000) (0.133, 0.220) | 0.295  (0.032) (0.000) (0.231, 0.358) | 0.260  (0.036) (0.000) (0.190, 0.330) |
|  | **Unable** | 0.051  (0.009) (0.000)  (0.033, 0.068) | 0.026  (0.005) (0.000)  (0.016, 0.037) | 0.059  (0.014) (0.000)  (0.032, 0.085) | 0.049  (0.012) (0.000) (0.026, 0.073) | 0.088  (0.021) (0.000) (0.047, 0.129) | 0.079  (0.020) (0.000) (0.040, 0.117) |
|  |  |  |  |  |  |  |  |
| **Self Care** | **None** | -0.268  (0.013) (0.000)  (-0.294, -0.242) | -0.134  (0.020) (0.000)  (-0.173,-0.096) | -0.309  (0.032) (0.000)  (-0.371, -0.247) | -0.264  (0.037) (0.000)  (-0.337, -0.191) | -0.484  (0.055) (0.000)  ( -0.592, -0.377) | -0.494  (0.067) (0.000)  (-0.625, -0.363) |
|  | **Slight** | 0.109  (0.009) (0.000)  (0.091, 0.126) | 0.051  (0.008) (0.000)  (0.035, 0.067) | 0.089  (0.011) (0.000)  (0.067, 0.111) | 0.074  (0.012) (0.000) (0.050, 0.098) | 0.104  (0.016) (0.000) (0.073, 0.135) | 0.105  (0.018) (0.000) (0.070, 0.140) |
|  | **Moderate** | 0.089  (0.009) (0.000) (0.071, 0.108) | 0.045  (0.008) (0.000)  (0.030, 0.061) | 0.114  (0.016) (0.000)  (0.082, 0.146) | 0.096  (0.017) (0.000) (0.063, 0.129) | 0.189  (0.030) (0.000) (0.130, 0.247) | 0.192  (0.034) (0.000) (0.126, 0.259) |
|  | **Severe** | 0.040  (0.007) (0.000)  (0.026, 0.055) | 0.022  (0.005) (0.000)  (0.012, 0.032) | 0.059  (0.013) (0.000)  (0.034, 0.083) | 0.051  (0.012) )0.000) (0.028, 0.074) | 0.104  (0.024) (0.000) (0.057, 0.152) | 0.107  (0.026) (0.000) (0.057, 0.158) |
|  | **Unable** | 0.030  (0.007) (0.000) (0.016, 0.044) | 0.016  (0.004) (0.000)  (0.008, 0.025) | 0.048  (0.012) (0.000)  (0.024, 0.072) | 0.043  (0.011) (0.000) (0.020, 0.0650 | 0.088  (0.023) (0.000) (0.042, 0.133) | 0.089  (0.024) (0.000) (0.041, 0.137) |
|  |  |  |  |  |  |  |  |
| **Usual Activities** | **None** | -0.431  (0.013) (0.000)  (-0.456,-0.405) | -0.234  (0.025) (0.000)  (-0.283,-0.186) | -0.360  (0.025) (0.000)  (-0.408, -0.311) | -0.304  (0.032) (0.000)  (-0.367,-0.241) | -0.479  (0.029) (0.000)  (-0.537, -0.422) | -0.436  (0.042) (0.000)  (-0.518, -0.355) |
|  | **Slight** | 0.125  (0.008) (0.000)  (0.109, 0.141) | 0.063  (0.009) 0.000)  (0.046, 0.079) | 0.005  (0.007) (0.480)  (-0.009, 0.020) | 0.002  (0.006) (0.711)  (-0.010, 0.015) | -0.038  (0.017, 0.023)  ( -0.071, -0.005) | -0.035  (0.015) (0.021)  (-0.066, -0.005) |
|  | **Moderate** | 0.128  (0.009) (0.000)  (0.111, 0.146) | 0.070  (0.008 (0.000)  (0.054, 0.086) | 0.096  (0.011) (0.000)  (0.075, 0.117) | 0.081  (0.011) (0.000) (0.059, 0.102) | 0.108  (0.016) (0.000) (0.076, 0.140) | 0.098  (0.016) (0.000) (0.066, 0.130) |
|  | **Severe** | 0.093  (0.009) (0.000)  (0.075, 0.110) | 0.053  (0.007) (0.000)  (0.039, 0.067) | 0.128  (0.015) (0.000)  (0.098, 0.158) | 0.109  (0.016) (0.000) (0.078, 0.139) | 0.174  (0.024) (0.000) (0.127, 0.220) | 0.158  (0.025) (0.000) (0.110, 0.206) |
|  | **Unable** | 0.085  (0.010) (0.000)  (0.065, 0.105) | 0.049  (0.008) (0.000)  (0.034, 0.064) | 0.131  (0.019) (0.000)  (0.093, 0.169) | 0.112  (0.019) (0.000) (0.076, 0.148) | 0.236  (0.034) (0.000) (0.169, 0.303) | 0.216  (0.035) (0.000) (0.148, 0.283) |
|  |  |  |  |  |  |  |  |
| **Pain / Discomfort** | **None** | -0.491  (0.018) (0.000)  (-0.526,-0.457) | -0.292  (0.029) (0.000)  (-0.349,-0.235) | -0.295  (0.022) (0.000)  (-0.337, -0.252) | -0.271  (0.026) (0.000)  (-0.322, -0.220) | -0.293  (0.024) (0.000)  (-0.341, -0.245) | -0.261  (0.031) (0.000)  (-0.322, -0.200) |
|  | **Slight** | 0.092  (0.010) (0.000)  (0.073, 0.112) | 0.052  (0.008) (0.000)  (0.036, 0.069) | -0.088  (0.011) (0.000)  (-0.108, -0.067) | -0.085  (0.011) (0.000)  (-0.106, -0.064) | -0.145  (0.018) (0.000)  (-0.180, -0.111) | -0.133  (0.019) (0.000)  ( -0.170, -0.096_ |
|  | **Moderate** | 0.201  (0.010) (0.000)  (0.181, 0.220) | 0.119  (0.012) (0.000)  (0.095, 0.143) | 0.096  (0.012) (0.000)  (0.071, 0.120) | 0.088  (0.013) (0.000) (0.062, 0.113) | 0.062  (0.018) (0.001) (0.027, 0.098) | 0.055  (0.017) (0.001) (0.022, 0.088) |
|  | **Severe** | 0.145  (0.011) (0.000)  (0.124, 0.166) | 0.090  (0.010) (0.000)  (0.070, 0.109) | 0.200 (0.019) (0.000)  (0.162, 0.238) | 0.188  (0.021) (0.000) (0.147, 0.229) | 0.259  (0.029) ( 0.000 (0.203, 0.316 | 0.235  (0.032) (0.000) (0.172, 0.298) |
|  | **Extreme** | 0.054  (0.009) (0.000)  (0.037, 0.071) | 0.032  (0.006) (0.000)  (0.020, 0.044) | 0.087  (0.016) (0.000)  (0.055, 0.118) | 0.080  (0.016) (0.000) (0.049, 0.111) | 0.116  (0.023) (0.000) (0.071, 0.161) | 0.104  (0.022) (0.000) (0.061, 0.147) |
|  |  |  |  |  |  |  |  |
| **Anxiety / Depression** | **None** | -0.298  (0.019) (0.000)  (-0.336, -0.261) | -0.250  (0.030) (0.000)  (-0.309,-0.190) | -0.295  (0.022) (0.000)  (-0.337, -0.252) | -0.322  (0.035) (0.000)  (-0.391, -0.253) | -0.489  (0.043) (0.000)  (-0.573, -0.406) | -0.470  (0.054) (0.000)  (-0.576,-0.363) |
|  | **Slight** | 0.114  (0.009) (0.000)  0.096, 0.132) | 0.094  (0.012) (0.000)  (0.070, 0.119) | -0.088  (0.011) (0.000)  (-0.108, -0.067)) | 0.075  (0.011) (0.000) (0.053, 0.096) | 0.084  (0.016) (0.000) (0.054, 0.115) | 0.080  (0.016) (0.000) (0.049, 0.111) |
|  | **Moderate** | 0.137  (0.011) (0.000)  (0.115, 0.160) | 0.115  (0.015) (0.000)  (0.086, 0.145) | 0.096  (0.012) (0.000)  (0.071, 0.120) | 0.176  (0.023) (0.000) (0.130, 0.221) | 0.279  (0.035) (0.000) (0.211, 0.347) | 0.267  (0.039) (0.000) (0.190, 0.344) |
|  | **Severe** | 0.032  (0.006) (0.000)  (0.020, 0.044) | 0.027  (0.006) (0.000)  (0.016, 0.039) | 0.200  (0.019) (0.000)  (0.162, 0.238) | 0.044  (0.012) (0.000) (0.021, 0.067) | 0.075  (0.020) (0.000) (0.036, 0.115) | 0.073  (0.020) (0.000) (0.033, 0.113) |
|  | **Extreme** | 0.015  (0.005) (0.001)  (0.006, 0.024) | 0.013  (0.004) (0.002)  (0.005, 0.020) | 0.087  (0.016) (0.000)  (0.055, 0.118) | 0.027  (0.010) (0.004) (0.009, 0.046) | 0.051  (0.017) (0.004) (0.017, 0.085) | 0.049  (0.017) (0.004) (0.015, 0.083) |
| **N** |  | 1495 | 1495 | 610 | 610 | 480 | 480 |

**Notes:** This table presents partial effect estimates for *Multimorbidity* from a series of ordered probit (OPROB) model specifications. Partial Effect Estimate, Standard Error (SE), P-Value, 95% Confidence Interval.

**Source:** Analysis of data from the Irish EQ-5D-5L Survey, 2015/16 (59), and data from the SPPiRE randomised controlled trial (60).
